# Supplementary material for: High HER2 Intratumoral Heterogeneity Is a Predictive Factor for Poor Prognosis in Early-Stage and Locally Advanced HER2-Positive Breast Cancer
Source: Cancers (Basel). 2024 Mar 5;16(5):1062. doi: 10.3390/cancers16051062 (PMC10930968; doi:10.3390/cancers16051062)
Supplement: Supplementary file 1 [file cancers-16-01062-s001.zip › cancers-2838563-supplementary.pdf]

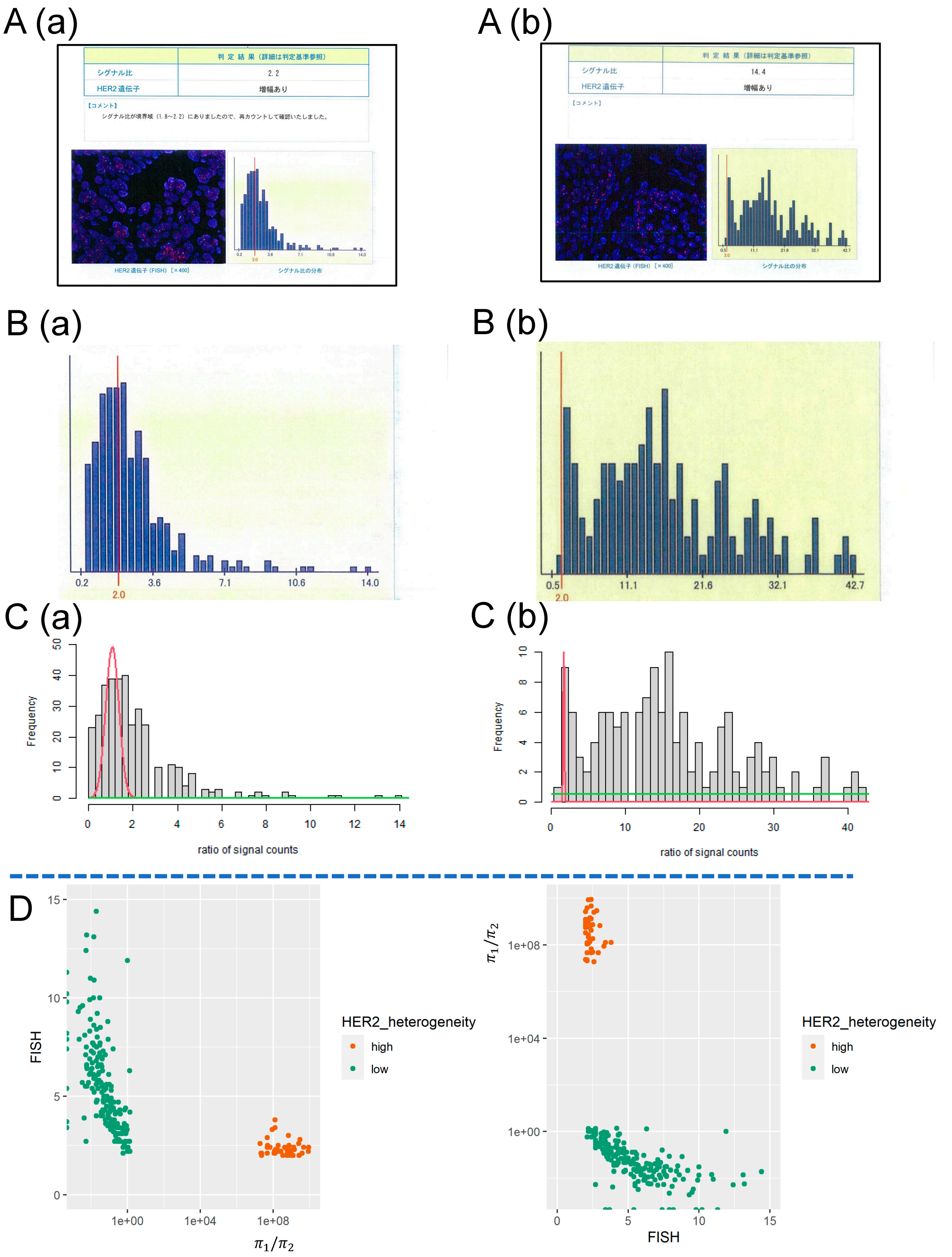

Figure S1. Procedure for imaging a diagnostic report. Two representative cases in which imaging a diagnostic report of HER2 FISH signals (A), histogram of HER2 FISH signals (B), and fitting by a mixture of Gaussian distribution (C) classified the cases into the HH (a) and LH group (b). HER2 FISH signals and ratio of mixtures (π1/π2) in the total sample of patients with HER2-positive breast cancer (D).

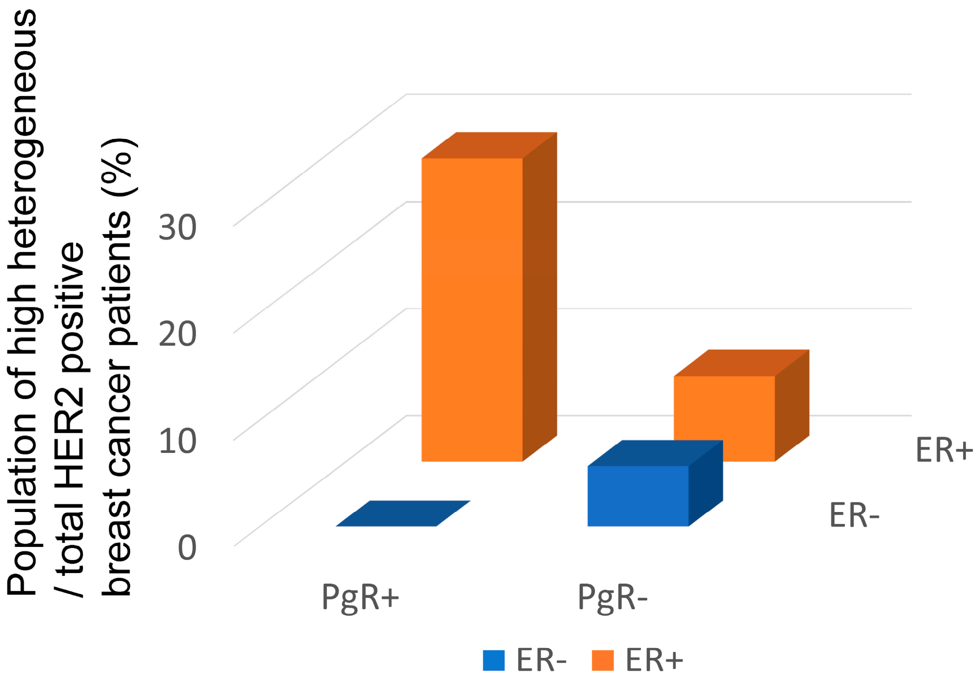

Figure S2. Relationship between the population of patients with highly heterogeneous/total HER2-positive breast cancer (percentage) and ER/PgR status (n = 251). The ER+ and PgR+ tumors demonstrated the highest frequency of HER2 heterogeneity (ER+ and PgR+: 28% [28/99], ER and PgR−: 8% [6/75], ER+ and PgR−: 6% [4/70], and ER− and PgR+: 0% [0/7]).

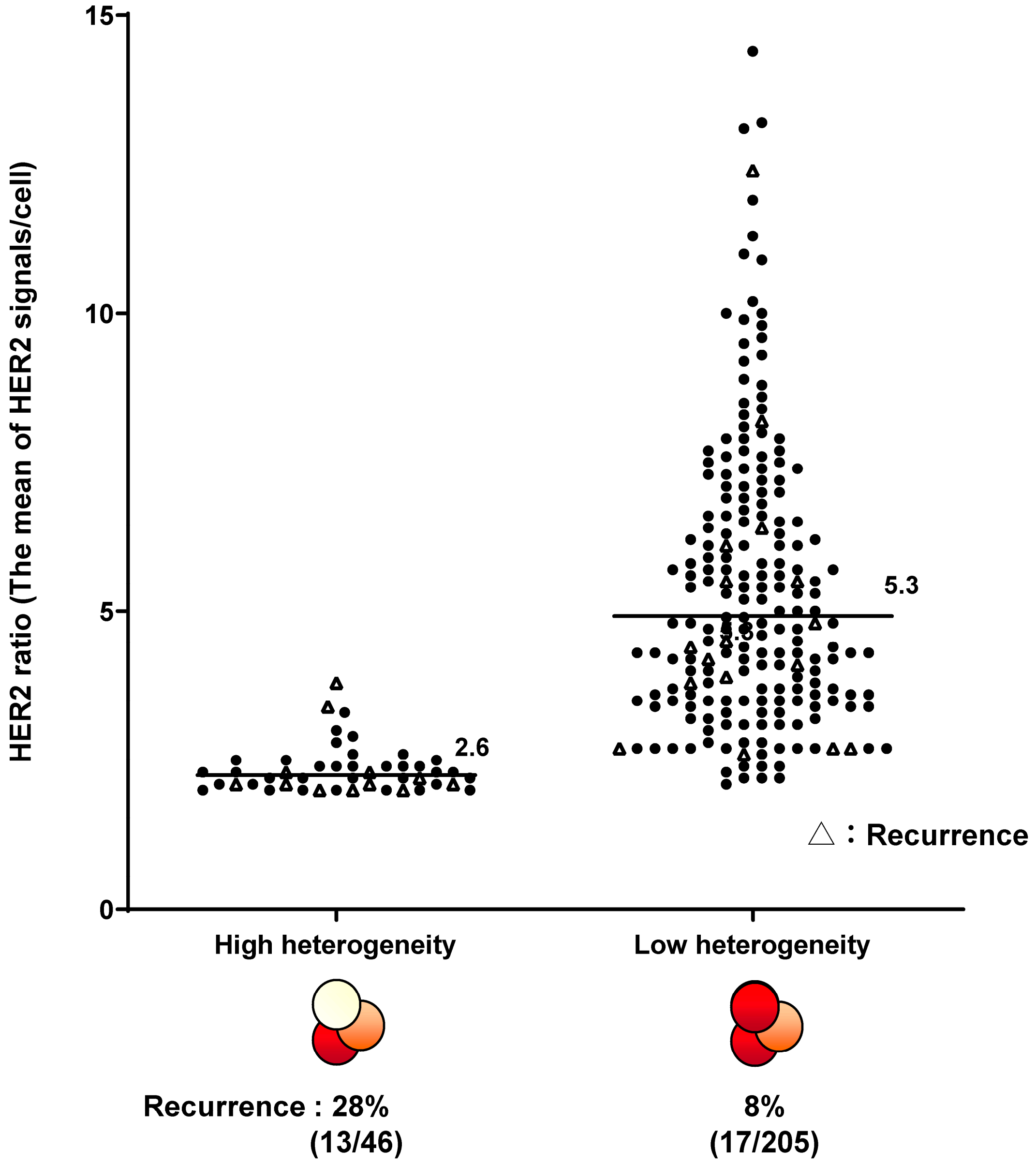

Figure S3. Relationship between the HER2 ratio (the mean HER2 signals/cell) and HER2 intratumoral heterogeneity. The HER2 ratio was compared between tumors with high and low heterogeneity (p-value, Mann–Whitney U-test; bars, median; patients experiencing. disease recurrence: white triangles; and patients with no recurrence: black dots).

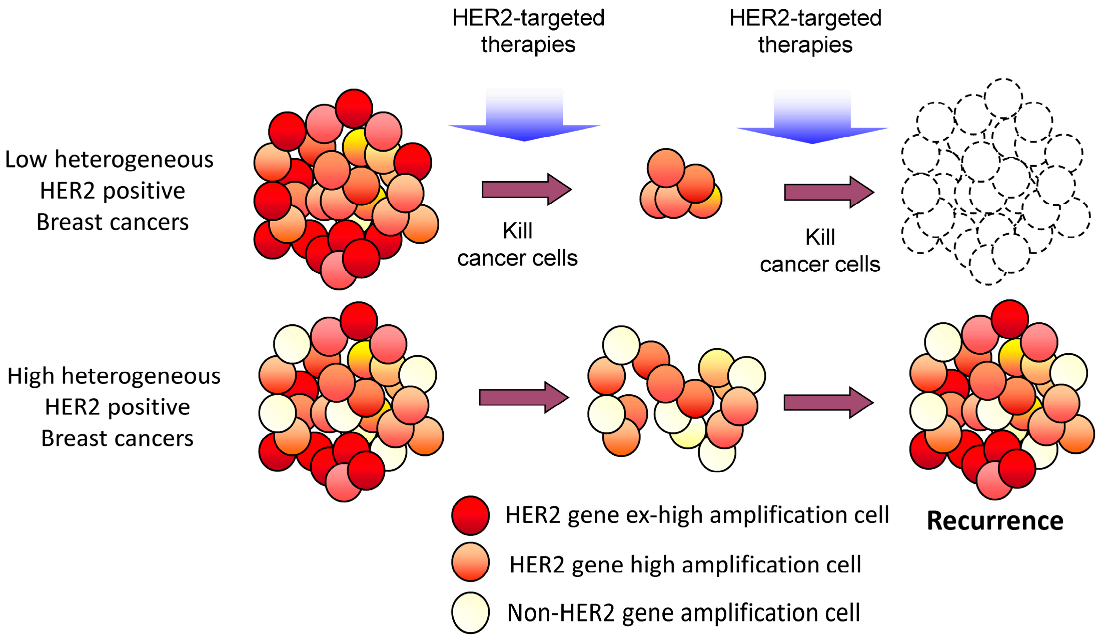

Figure S4. Schematic diagram of the effect of HER2-targeted therapies in HER2 intratumoral heterogeneity. High levels of heterogeneity of HER2 were clinically significant for poor prognosis for resistance to adjuvant therapy with HER2-targeted therapies in primary breast cancers.
